# Punchline with(out) purpose: Integrating research on instructional humour and seductive details

**DOI:** 10.1111/bjep.70080

**Published:** 2026-03-29

**Authors:** Lisa Bender, Alexander Renkl, Tino Endres

**Affiliations:** ^1^ University of Freiburg Freiburg Germany; ^2^ Faculty of Psychology UniDistance Suisse Brig Switzerland; ^3^ University of Zurich Zurich Switzerland

**Keywords:** cognitive load, instructional humour, mental effort, relevance, seductive details, situational interest, teacher humour

## Abstract

**Introduction:**

We integrated research on instructional humour and seductive details to investigate when affiliative course‐related humour is effective or rather ineffective for learning. We assumed that instructional humour without a cognitive function (irrelevant humour) would have detrimental effects on learning performance resembling the seductive details effect, whereas humour with a cognitive function (e.g., humorous analogies illustrating the learning content) would foster performance.

**Methods:**

Our hypotheses were tested in two between‐subjects studies. Study 1 was conducted in the laboratory with 115 adults (*M*_age_ = 24.71); Study 2 was conducted in a German high school with 151 youths (*M*_age_ = 12.21). In both studies, participants learned from a narrated slideshow in one of four conditions: the slideshow either contained irrelevant humour, relevant humour, relevant (non‐humorous) examples or no additional elements (control). We then assessed learning performance, cognitive load and affective‐motivational aspects.

**Results:**

In study 1, the relevant‐humour group indeed outperformed the irrelevant‐humour group in terms of transfer. We replicated this effect in study 2 for recall performance. Our comparison to the control group without additional elements was less conclusive: The irrelevant‐humour group scored worse than the control group in study 1 (i.e., seductive details effect), but not in study 2. Moreover, the relevant‐humour group did not clearly outperform the control group in either of the studies.

**Conclusion:**

It may be safer to avoid irrelevant instructional humour (even when course‐related) because of the risk of a detrimental seductive details effect. Further research is needed with respect to the cognitive and affective‐motivational effectiveness of relevant instructional humour.

## BACKGROUND

When creating and delivering their learning units, instructors often face the question of whether to include jokes, humorous anecdotes or fun facts. The evidence is contradictory on the effects of such adjuncts. On the one hand, survey‐based research on course‐related teacher humour revealed positive associations with cognitive and affective‐motivational outcomes (e.g., Bieg & Dresel, 2018; Wanzer et al., 2010). On the other hand, seductive details research suggests that augmenting learning material with interesting texts and/or pictures that are tangentially related to but irrelevant for the learning goal can hamper learning (e.g., Garner et al., 1989; Sundararajan & Adesope, 2020). One explanation for these inconsistent findings refers to the various functions that humorous elements fulfil in a learning unit (e.g., Cazes et al., 2024; Wanzer et al., 2010). For example, some humorous elements may be both entertaining (i.e., affective‐motivational function) and illustrate the learning content or add relevance (i.e., cognitive function). Such relevant humour could enable *and* motivate deep, effortful processing (see e.g., Eitel et al., 2025; Wanzer et al., 2010). In contrast, humorous elements lacking an additional cognitive function might act as a detrimental seductive detail because they interfere with students' processing of the relevant content (Harp & Mayer, 1998; see chapter ‘Research on Seductive Details’ for detailed information). Two studies investigated when instructional humour is effective or ineffective for learning by focussing specifically on how humour's affective‐motivational and cognitive functions influence learning. The present work thereby aims to bring the related research fields of teacher humour and seductive details closer together.

### Research on (course‐related) instructional humour

Instructional humour (also referred to as teacher humour) is a fairly broad construct ranging from jokes or witty comments to comics, pictures, funny voices or sounds (e.g., Banas et al., 2011; Frymier et al., 2008; Martin, 2007; Robinson et al., 2024). Likewise, researchers view the functions and effects of instructional humour as quite multidimensional (e.g., Bieg & Dresel, 2018; Frymier et al., 2008; Martin et al., 2003). The focus of the present paper is affiliative humorous messages from an instructor (e.g., jokes and funny anecdotes). Such humour might mainly be incorporated to amuse and motivate students, facilitate relationships or reduce tension, but it could also serve cognitive functions (e.g., Banas et al., 2011; Bieg & Dresel, 2018; Martin et al., 2003).

Survey studies have revealed positive associations between affiliative humour and different instructional dimensions (Banas et al., 2011; Bieg et al., 2019; Bieg & Dresel, 2018; Wanzer et al., 2010). For example, a survey with 985 secondary school students in Germany revealed positive relationships between perceived teacher humour and socio‐emotional and motivational dimensions on the one hand (e.g., perceived teacher–student relationship, interestingness and enjoyment of instruction) and cognitive dimensions on the other hand (perceived clarity of instruction and elaboration; Bieg & Dresel, 2018). Noteworthy, these positive relationships were only apparent for humour that is *related* to the course content (i.e., has a thematic connection to the topic in class). Course‐unrelated humour proved to be not at all or even negatively associated with such outcomes (e.g., Bieg & Dresel, 2018; Frymier et al., 2008). This pattern of results was also supported by an experimental study comparing students' ratings of videos displaying a teaching unit containing four different types of humour or no humour (Bieg et al., 2026).

Learning experiments comparing the effects of course‐related affiliative humour on learning performance to no‐humour conditions, however, yielded inconsistent evidence. While some supported the positive effects on learning outcomes observed in survey studies, others revealed no effect or even reported negative effects (e.g., Bolkan et al., 2018; Cazes et al., 2024; Houser et al., 2007; Ziv, 1988). For example, while humorous examples and anecdotes in an online lecture improved recall performance in a study by Garner (2006), Houser et al. (2007) observed no such effect. In a study by Bolkan et al. (2018), students learning from a written lecture with humorous examples showed worse recall and transfer performance than a control group reading the lesson with neutral examples. These results indicate that course‐related instructional humour might not be beneficial per se, but could also have negative effects.

### Research on seductive details

Although the research field of seductive details developed rather separately from instructional humour research (for an exception, see, e.g., Dorambari, 2022; Robinson, 2022), the two fields are related. Similar to course‐related instructional humour, seductive details usually consist of entertaining information that is tangentially related to the learning content (e.g., Eitel et al., 2022; Harp & Mayer, 1998). Moreover, seductive details occasionally comprise humorous elements to increase their entertainment value or interestingness (e.g., Bender et al., 2021a; Gardner et al., 2016; Robinson, 2022; Sitzmann & Johnson, 2014). Hence, the research field of seductive details might provide some insights that are also transferable to instructional humour and vice versa. So far, researchers have investigated seductive text passages or narrations (e.g., Garner et al., 1989; Ozdemir & Doolittle, 2015), decorative pictures (e.g., Lenzner et al., 2013; Magner et al., 2014), as well as combinations thereof (e.g., Bender et al., 2021a; Harp & Mayer, 1998). Overall, meta‐analyses have demonstrated small to medium negative effects of such details on learning performance, although the negative effects have appeared specifically among learners possessing poorer cognitive prerequisites, such as less prior knowledge and inferior academic achievement (e.g., Bender et al., 2021b; Korbach et al., 2016; Sundararajan & Adesope, 2020). This finding motivated the general recommendation to avoid seductive. The explanation for this negative effect originates in the cognitive load theory (Sweller et al., 2011) and the cognitive theory of multimedia learning (Mayer, 2021). Both theories assume that learning from complex visuospatial and verbal material (i.e., multiple representations) already places high demands on students' limited working memory capacity (i.e., intrinsic cognitive load; Sweller et al., 2011). Seductive details can place an additional unnecessary load on the cognitive system because they are irrelevant for reaching the learning goal (i.e., extraneous cognitive load). This increase in extraneous load, in turn, hampers the processing of relevant content, resulting in a poorer learning performance. Moreover, seductive details can even evoke specific detrimental cognitive processes (Harp & Mayer, 1998; Mayer, 2021): they might distract the learners' attention away from the relevant learning content, they might disrupt the flow of organizing and understanding the learning content, or they might trigger distracting thoughts and inappropriate prior knowledge, thereby diverting the construction of a mental model.

Hence, although seductive details may be related to the to‐be‐learned topic (as is the case for course‐related affiliative humour), they still hamper learning because they are irrelevant to the actual learning goal. The same could apply likewise to instructional humour: Course‐related affiliative humour might hamper learning in cases when it is irrelevant to the learning goal. In contrast, beneficial effects on learning performance are possible when the course‐related humour is relevant. The instructional humour processing theory (Wanzer et al., 2010) provides a theoretical framework for this idea.

### Integrating the research fields: Instructional humour processing theory

According to the instructional humour processing theory (Wanzer et al., 2010), instructional humour can inspire effortful processing of the learning content and thereby improve the learning performance, but only if two requirements are met. First, students should be able to perceive the humour and consider it appropriate to ensure that it evokes amusement and/or laughter (*affective‐motivational function*). The humour can thereby increase students' motivation and mental effort (see also Moreno, 2005). Similar ideas have already been considered and tested in the research field of emotional design. In this research, some studies have demonstrated that designing learning material with emotionally pleasing colours, forms, characters and narratives led to positive feelings and greater situational interest, motivation and mental effort (e.g., Endres et al., 2020; Endres, Vössing, et al., 2025; Mayer & Estrella, 2014; Stark et al., 2018).

However, the instructional humour processing theory also suggests that the affective‐motivational function alone might not suffice for instructional humour to convey positive effects on learning performance. As a second function, the humorous information should also help students to process the learning content, that is, by clarifying the content or demonstrating its relevance (i.e., *cognitive function*). This function could be achieved via humorous examples, anecdotes, or metaphors specifically illustrating the content to be learned (e.g., Garner, 2006; Ziv, 1988). For example, in the study by Garner (2006), relevant humorous stories, examples and metaphors in a recorded statistics lecture led to a more positive evaluation of the learning unit and of the instructor, as well as better recall of the relevant information. In contrast, if instructional humour only comprises the affective‐motivational function and fails to help students process the content or add relevance, it might serve instead as a seductive detail because it comprises irrelevant information that hampers learning.

Similar assumptions can be drawn from the FoRe‐Squares model (Eitel et al., 2025), suggesting that an instruction should comprise both motivational techniques increasing reward *and* cognitive techniques strengthening focus and support in order to enable and motivate active goal‐directed processing. Hence, only relevant humour might improve learning since it both increases reward (thanks to its humorous nature) and facilitates support (due to its relevance). Relying on these assumptions and in line with the argumentation by Banas et al. (2011) and Cazes et al. (2024), we conclude that the presence or absence of a cognitive function (i.e., the [ir]relevance of the humour) may be able to – at least in part – explain inconsistent findings on teacher humour from previous studies.

### Present research and hypotheses

**FIGURE 1:**
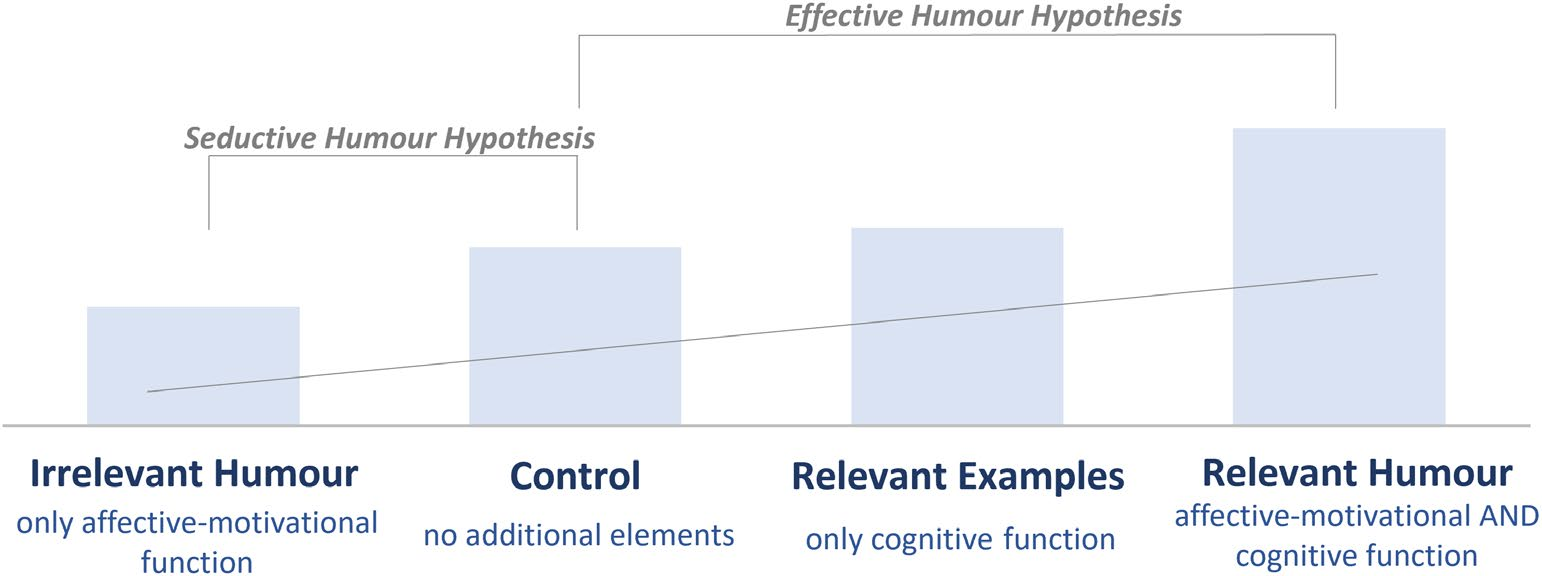
Assumed pattern of results.

**FIGURE 2:**
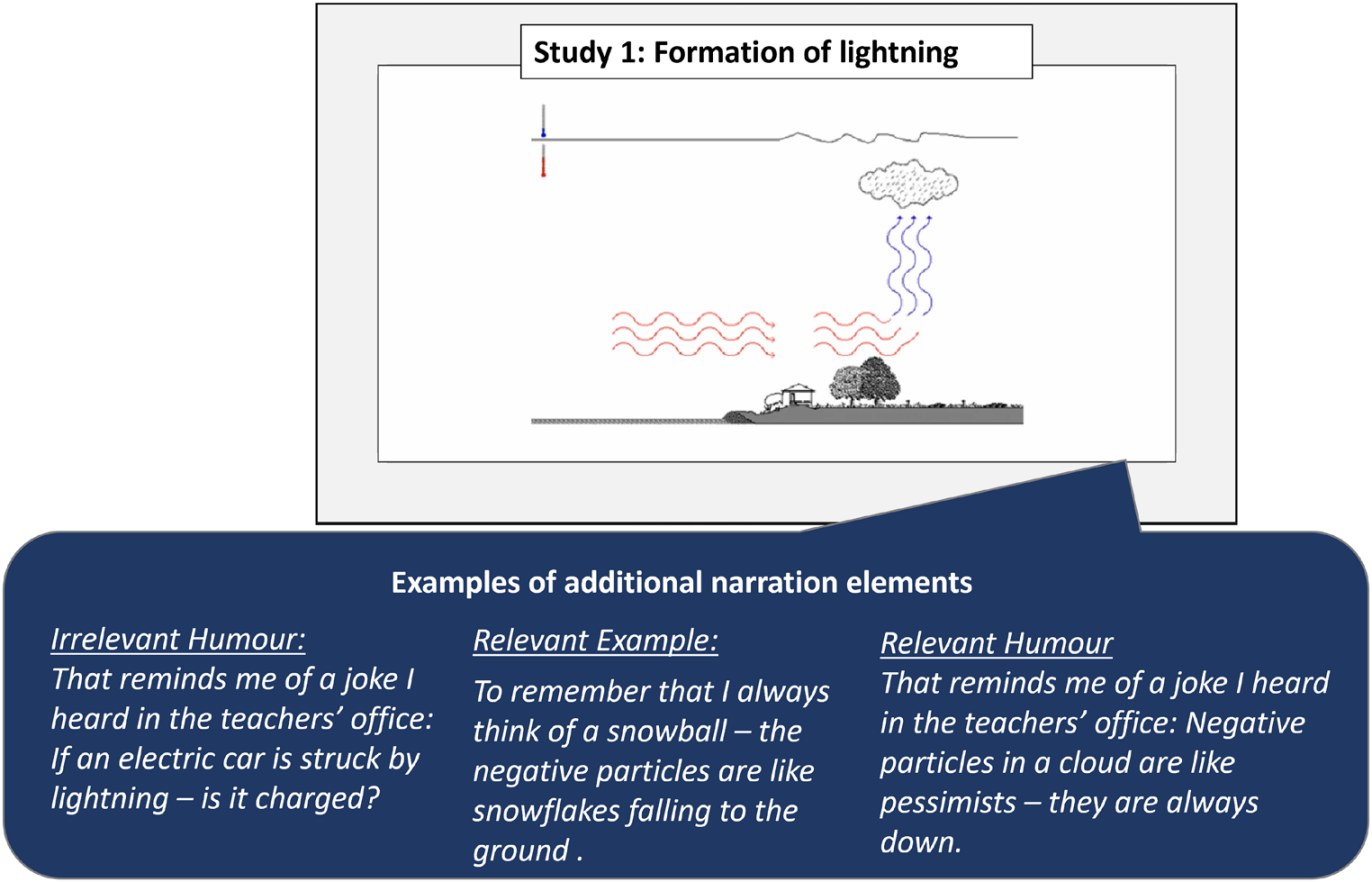
Screenshot of the learning material (narrated slides) and the additional narration elements in the four experimental groups of Study 1.

In our studies, we integrated research on instructional humour and seductive details to investigate when course‐related instructional humour is detrimental or beneficial for learning outcomes (research question 1). Moreover, we investigated the effects of instructional humour on cognitive (research question 2) and affective‐motivational processing (research question 3). For this purpose, we compared the effects of different versions of a slide show (see Figures 1, 2 and 6): One version contained *irrelevant humour* (i.e., only affective‐motivational function), another version contained *relevant humour* (i.e., affective‐motivational *and* cognitive function). Moreover, as comparison groups, we added a version containing *relevant* (*non‐humorous*) *examples* (i.e., only cognitive function) and a *control group* with no additional elements.

#### Research question 1

We assumed that irrelevant humour (i.e., an affective‐motivational function only) in a slide show would hamper learning compared to a slide show without humour, because the irrelevant humour would add unnecessary information that might increase extraneous load on the students' working memory, similar to a seductive details effect (*seductive‐humour hypothesis*, H1.1). In contrast, we assumed that relevant humour (i.e., cognitive and affective‐motivational function) would lead to better learning outcomes compared to no humour (*effective humour hypothesis*, H1.2), because it inspires students to process the learning content more deeply and effortfully without adding irrelevant information.

Lastly, we expected that relevant examples (i.e., cognitive function only) would result in better learning outcomes compared to the control group. However, we still expected the relevant‐examples group to score worse than the relevant‐humour group, because the examples lack the affective‐motivational function (no humour), increasing mental effort (e.g., Eitel et al., 2025; Moreno, 2005). Overall, we therefore expected the following linear pattern: irrelevant humour < control < relevant examples < relevant humour (see Figure 1).

#### Research question 2

In line with the cognitive load theory (Sweller et al., 2011) and past research on seductive details, we assumed that the negative effect of irrelevant humour compared to no humour would be mediated by higher extraneous cognitive load (H2.1). Moreover, in line with the instructional humour processing theory (Wanzer et al., 2010), we assumed that the positive effect of relevant humour compared to no humour would be mediated by greater mental effort (H2.2., see also Moreno, 2005).

#### Research question 3

In addition to the effects on learning outcomes, we also tested the instructional humour processing theory's assumptions. We, therefore, expected that irrelevant and relevant instructional humour would improve affective‐motivational processing, that is, higher ratings of situational interest (H3.1), instructor likeability (H3.2) and learning atmosphere (H3.3) compared to the groups with (non‐humorous) relevant examples or no additional elements.

To test the above hypotheses in different contexts, we conducted one laboratory study with an adult sample and another school study with a younger sample. Specifically, we decided for both, an adult and a student sample, due to past seductive details studies showing positive affective‐motivational effects specifically for younger learners (average age of 13–15 years; e.g., Magner et al., 2014; Lenzner et al., 2013; Wang & Adesope, 2016), but not as consistently for adult learners (e.g., Bender et al., 2021a, 2021b; Park et al., 2015). Hence, it is possible that the effects of humour are different for younger learners than for adult learners. In both studies, we used meaningful learning materials on natural phenomena (study 1: formation of lightning; study 2: solar and lunar eclipses) and, therefore, controlled for cognitive prerequisites that might influence learning performance (see Sweller et al., 2011). In study 2, we additionally aimed for higher power due to inconclusive findings in study 1.

## STUDY 1

### Method

#### Participants and design

An a *priori* power‐analysis with G*Power (Faul et al., 2007) revealed that to ensure the assumed medium effect size (Cazes et al., 2024; Sundararajan & Adesope, 2020), our experimental design would require a sample size of 101 participants to detect a statistically significant linear contrast in a contrast analysis (directed hypothesis, α‐level *p* = .05, Cohen's *f* = .25, power 80%). We recruited participants via a participant database, flyers and announcements on bulletin boards. Undergraduate psychology students received course credit (1 h) for participating.

We collected data from 123 participants to compensate for potential drop‐outs. In accordance with our study requirements, we excluded four participants from our analyses whose German language skills were not up to the required level and four participants with expertise in physics (our learning material's topic). We obtained a final sample of 115 participants (*M*_age_ = 24.71) that consisted of 87.8% undergraduates, 8.7% employees and 3.5% with other occupations.

This study followed a one‐factorial between‐subjects design with four experimental groups to which we assigned participants randomly (see Figures 1 and 2). Participants learned from a narrated slide show that contained narrated relevant humour (*n* = 32), narrated irrelevant humour (*n* = 27), narrated relevant examples (*n* = 28) or no additional elements (*n* = 28).

#### Materials and experimental manipulations

A narrated slide show about the formation of lightning served as a learning material. The narration consisted of slightly adapted texts from studies by Harp and Mayer (1998) and Eitel et al. (2019), and was performed by a middle‐aged male narrator. The text was of appropriate complexity for our age group (German version of Flesch–Kincaid score = 51.69; Lenhard & Lenhard, 2014). The accompanying slides showed four schematic illustrations (also adapted from Harp & Mayer, 1998, and Eitel et al., 2019), in which the important components were highlighted with circles and arrows to optimize participants' processing of the slide show (see signalling principle; van Gog, 2021). The base video lasted 4 min 24 s and could not be paused by participants.

In three of our four experimental groups, the narration contained additional narrated elements. In the relevant‐humour group, the narrator made seven jokes throughout the learning unit that illustrated the content via humorous analogies (see Figure 1 for an example). In the irrelevant‐humour group, the narrator also made seven jokes, but those jokes—although related to the topic of lightning—were not relevant. Lastly, in the relevant‐examples group, the narrator provided seven examples that illustrated the content via analogies but these were not humorous or funny. The additional elements in all three groups represented around 30% of the total narration; they were provided orally and inserted at the same points in the video. The experimental videos were approximately 1 min and 10 s longer than the base video.

In developing the jokes, we drew on the definition of jokes as containing a form of incongruity and pertinent humour techniques (e.g., Berger, 1976; Wanzer et al., 2010). We first tested the jokes and examples to check their funniness and cognitive function with a sample of 14 participants. A repeated‐measures contrast analysis revealed that participants rated the relevant (*M* = 3.19, SD = .82) and irrelevant jokes (*M* = 3.10, SD = .84) as funnier than the relevant examples (*M* = 1.97, SD = .87), *F*(1,13) = 35.77, *p* < .001 and ƞ^2^_p_ = 73. Moreover, they found that the relevant jokes (*M* = 3.66, SD = .74) and relevant examples (*M* = 3.70, SD = .76) promoted understanding more than the irrelevant jokes (*M* = 1.66, SD = .73), *F*(1,13) = 63.83, *p* < .001 and ƞ^2^_p_ = 83.

#### Measures

Unless indicated otherwise, all Likert self‐rating scales ranged from 1 ‘strongly disagree’ to 5 ‘strongly agree’.

##### Subjective prior knowledge

As a control variable potentially influencing learning performance, we assessed participants' subjective prior knowledge in metrology via six binary (yes/no) self‐rating items (e.g., ‘I know what a cold front is’) and one continuous self‐rating item (‘Please indicate how much you think you know about meteorology [weather]’; 5‐point Likert scale from 1 very low to 5 *very high*) adapted from Harp and Mayer (1998). The mean of the z‐standardized values served as a prior knowledge score (Cronbach's α = .76).

##### Spatial abilities

As a further cognitive prerequisite that might influence learning performance in our audio‐visual learning unit, we measured participants' spatial abilities (working memory substructure) with the first part of the paper folding test (VZ‐2; Ekstrom et al., 1976). On ten items, participants had to indicate what a squared piece of paper looks like while being unfolded. To perform the task correctly, participants must mentally imagine the transformation of the paper piece. This test lasted 3 min. We gave one point for each correct answer and subtracted one point for each false answer, leading to a score from −10 to 10 points.

##### Cognitive load

After learning, participants indicated their cognitive load via a 7‐item scale by Klepsch et al. (2017). On a 5‐point Likert scale (from 1 *not at all* to 5 *very much*) this scale assessed intrinsic cognitive load^1^ (two items, e.g., ‘This task was very complex.’, Cronbach's α = .66), extraneous cognitive load (three items, e.g., ‘The design of this task was unfavourable for learning.’, Cronbach's α = .81) and germane cognitive load (two items, e.g., ‘My point while dealing with the task was to understand everything correctly.’, Cronbach's α = .47) during the learning unit.

##### Mental effort

We implemented an adapted version of the mental effort item by Paas (1992; ‘I invested effort while working on this learning unit’; 5‐point Likert scale, 1 *not at all* to 5 *very much*). Due to a theoretical and empirical overlap between germane cognitive load and mental effort (*r* = .47, *p* < .001; Endres, Bender, et al., 2025; Klepsch & Seufert, 2021), we computed a composite score of the two measures, henceforth called mental effort (Cronbach's α = .62).

##### Affective‐motivational measures

As the first affective‐motivational measure, we assessed participants' situational interest (see Hidi & Renninger, 2006) via a scale by Endres et al. (2020, 5‐point Likert scales). The triggered situational interest subscale addressed participants' *feeling* towards the learning *material* (three items, e.g., ‘I find the design of the video entertaining.’, Cronbach's α = .88). The maintained situational interest scale addresses participants *feeling and value* towards the learning *content* (six items, e.g., ‘I find the topic presented in this video entertaining/useful.’, Cronbach's α = .82).^1^

Moreover, two self‐developed scales assessed how participants perceived the instructor's likability (5 items; e.g., ‘I find the instructor funny.’, Cronbach's α = .81) and the atmosphere throughout the learning unit (3 items; e.g., ’I enjoyed the learning unit.’, Cronbach's α = .85, 5‐point Likert scales).

##### Learning performance

We measured learning performance via a free recall task (‘Please write down everything you know about the formation of lightning’; Harp & Mayer, 1998). A rater who was blind to the experimental condition scored the answers according to a pre‐defined coding Scheme (0–12 points). For higher scores on this test, correct retention and basic comprehension of the main aspects were required. A second rater scored approximately 23% of the answers, resulting in good interrater‐reliability of ICC_2,1_ = .89 (two‐way random effects, absolute; Shrout & Fleiss, 1979). With Cronbach's α = .58, we obtained an acceptable internal consistency for heterogeneous constructs (Schmitt, 1996).

A multiple‐choice test with nine items (four to five answer options per item) served to assess transfer performance (e.g., ‘Why are clouds often seen in the sky, but no lightning strikes?’; Eitel et al., 2019). We gave one point for each correctly chosen answer option, leading to a score from 0 to 17 points. With Cronbach's α = .53, we obtained an acceptable internal consistency for heterogeneous constructs (Schmitt, 1996).

### Procedure

The experiment was conducted in the laboratory in sessions with up to 10 participants. Participants completed the study individually on a computer. After providing informed consent, they answered demographic questions (age, gender, first language, level of education, occupation, study program, semester, final school grade and physics courses at school and university). Participants then self‐rated their prior knowledge in meteorology and worked on the paper‐folding test. For the subsequent learning unit, participants were instructed to watch the video carefully because there would be questions on its content later on. Participants started the slideshow video themselves, but could not press pause. After the learning unit, participants self‐rated their perceived cognitive load, mental effort, situational interest, teacher likability, learning atmosphere and worked on the post‐test (free recall and transfer). We then debriefed them and thanked them for their participation. One session lasted about 30 min.

### Data analysis

We applied a three‐step approach for our analyses: First, we tested the comparability of our experimental groups with regard to control variables and checked the assumptions for our hypothesis tests (see OSF). Second, we decided on covariates. To avoid overly complex or biased analyses, we included potential covariates only in hypothesis testing when they indeed explained significant variance in the respective dependent variable (Field, 2024; Wildt & Ahtola, 1978). Third, we conducted the hypothesis tests: For the tests of our assumptions about learning performance (RQ1) and affective‐motivational processing (RQ3), we conducted multivariate, one‐factorial analyses of (co)variance with planned contrasts followed by LSD post‐hoc test in SPSS version 28 (alpha level of .05). For space and clarity reasons, we only report the results of the planned contrast tests (and not of the omnibus tests), since only those analyses refer directly to our hypotheses. Moreover, we conducted mediation analyses using the PROCESS tool version 4.3.1 (Hayes, 2022) to test for mediations via cognitive load and mental effort (RQ2). Follow‐up Bayesian analyses were conducted with JASP version 0.19.0. Our data can be accessed on OSF: https://osf.io/gnkf9.

### Results

#### Research question 1: Learning outcomes

**FIGURE 3:**
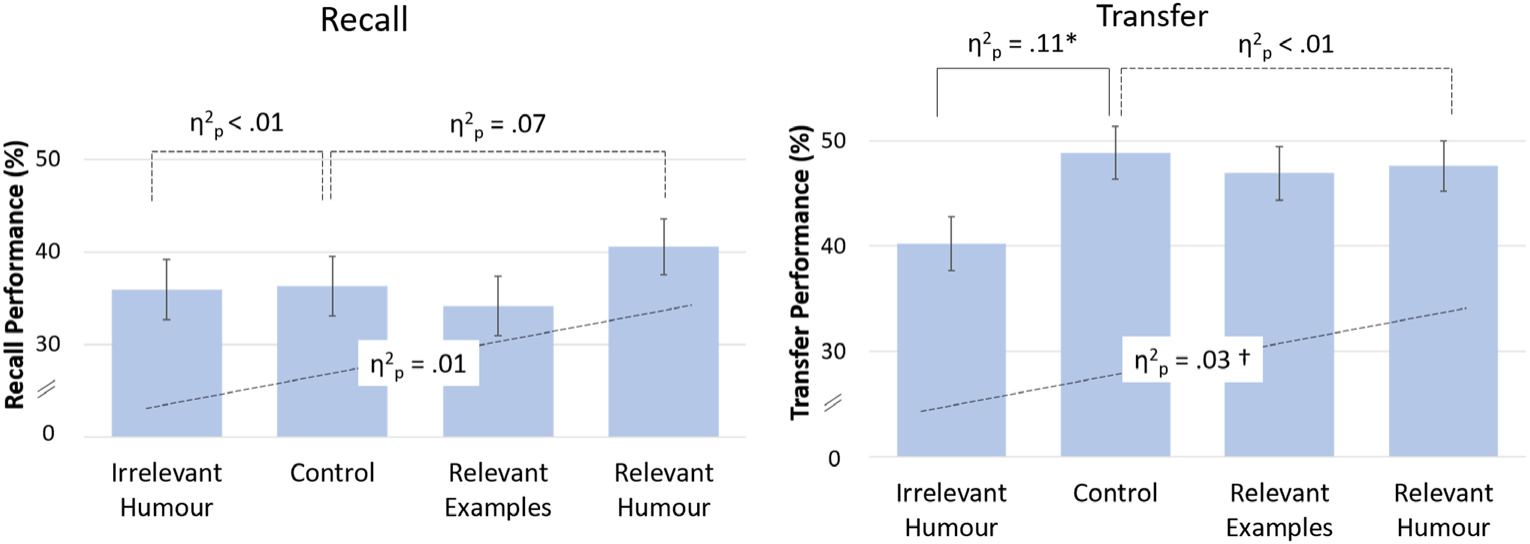
Recall and transfer performance in the four experimental conditions as a function of spatial abilities. Error bars indicate standard errors. ^†^ *p* < .10, *p < .05.

See Table 1 for descriptive values. To test our hypothesized linear pattern of results, we conducted a MANCOVA with a planned linear contrast. Recall and transfer performance served as dependent variables. Spatial abilities were used as control variable due to moderate correlations with recall (*r* = .26) and transfer (*r* = .28).^2^ The contrast analyses revealed no significant linear trend for recall, *F*(1,110) = .70, *p* = .404 and ƞ^2^_p_ = .01, and failed to reach the level of statistical significance for transfer performance, *F*(1,110) = 3.36, *p* = .069 and ƞ^2^_p_ = .03 (see Figure 3).

**TABLE 1:** Means (and standard deviations) of the main variables in the four experimental groups and condensed over all groups of study 1.

	Irrelevant humour	Control	Relevant examples	Relevant humour	Overall
Recall %	35.96 (14.25)	36.61 (17.76)	33.78 (17.94)	40.50 (19.09)	36.85 (17.40)
Transfer %	40.31 (11.84)	49.16 (14.25)	46.64 (14.22)	47.61 (14.67)	46.04 (14.05)
ECL [1; 5]	3.38 (.79)	2.81 (1.03)	2.62 (.91)	2.61 (1.01)	2.84 (.98)
ICL [1; 5]	3.56 (.73)	3.45 (.87)	3.39 (1.05)	3.34 (.86)	3.43 (.88)
Mental effort [1; 5]	3.60 (.76)	3.60 (.52)	3.85 (.62)	3.89 (.72)	3.74 (.67)
Triggered situational interest [1; 5]	2.56 (1.22)	2.25 (.81)	2.74 (1.02)	3.18 (1.13)	2.70 (1.10)
Maintained situational interest [1; 5]	3.44 (.80)	3.75 (.59)	3.68 (.63)	3.75 (.85)	3.66 (.73)
Instructor likability [1; 5]	3.25 (.79)	2.86 (.67)	3.44 (.70)	3.63 (.71)	3.31 (.76)
Learning atmosphere [1; 5]	3.01 (1.00)	3.25 (.76)	3.60 (.77)	3.73 (.74)	3.41 (.86)
Spatial ability [−10; 10]	4.70 (3.31)	4.89 (3.70)	4.39 (3.28)	4.63 (3.21)	4.65 (3.34)
Prior knowledge (z‐standardized)	−.18 (.35)	.08 (.72)	−.13 (.62)	.20 (.73)	.00 (.06)

Abbreviations: ECL, extraneous cognitive load; ICL, intrinsic cognitive load.

In line with the *seductive‐humour hypothesis* (H1.1.), post‐hoc comparisons revealed that the irrelevant‐humour group's transfer performance was significantly worse than both the control group's (*M*_diff_ = −8.63, *p* = .018, 95% CI [−15.76; −1.51]) and the relevant‐humour group's (*M*_diff_ = −7.40, *p* = .036, 95% CI [−13.82; −.50]). The difference from the relevant‐examples group failed to reach the level of statistical significance (*M*_diff_ = −6.70, *p* = .065, 95% CI [−13.82; .43]). Similar to previous studies (e.g., Bender et al., 2021a; Eitel et al., 2019), there were no such differences in recall performance, irrelevant humour vs. control: *M*_diff_ = −.40, *p* = .931, 95% CI [−9.46; 8.66]; irrelevant humour vs. relevant humour: *M*_diff_ = −4.64, *p* = .297, 95% CI [−13.42; 4.13]; irrelevant humour vs. relevant examples: *M*_diff_ = 1.76, *p* = .700, 95% CI [−7.29; 10.82].

In contrast to the *effective humour hypothesis* (H1.2), participants in the relevant‐humour group did not outperform the control group (recall: *M*_diff_ = 4.24, *p* = .335, 95% CI [−4.45; 19.93]; transfer: *M*_*diff*_ = −1.24, *p* = .721, 95% CI [−8.08; 5.60]). Following up on this insignificant finding, we additionally conducted a Bayesian ANCOVA comparing the relevant‐examples group to the control group to obtain evidence for the null hypothesis. The Bayes analysis revealed anecdotical evidence favouring the null hypothesis for recall performance (BF_01_ = 2.74) and moderate evidence for transfer (BF_01_ = 3.59).

#### Research question 2: Cognitive load and mental effort

**FIGURE 4:**
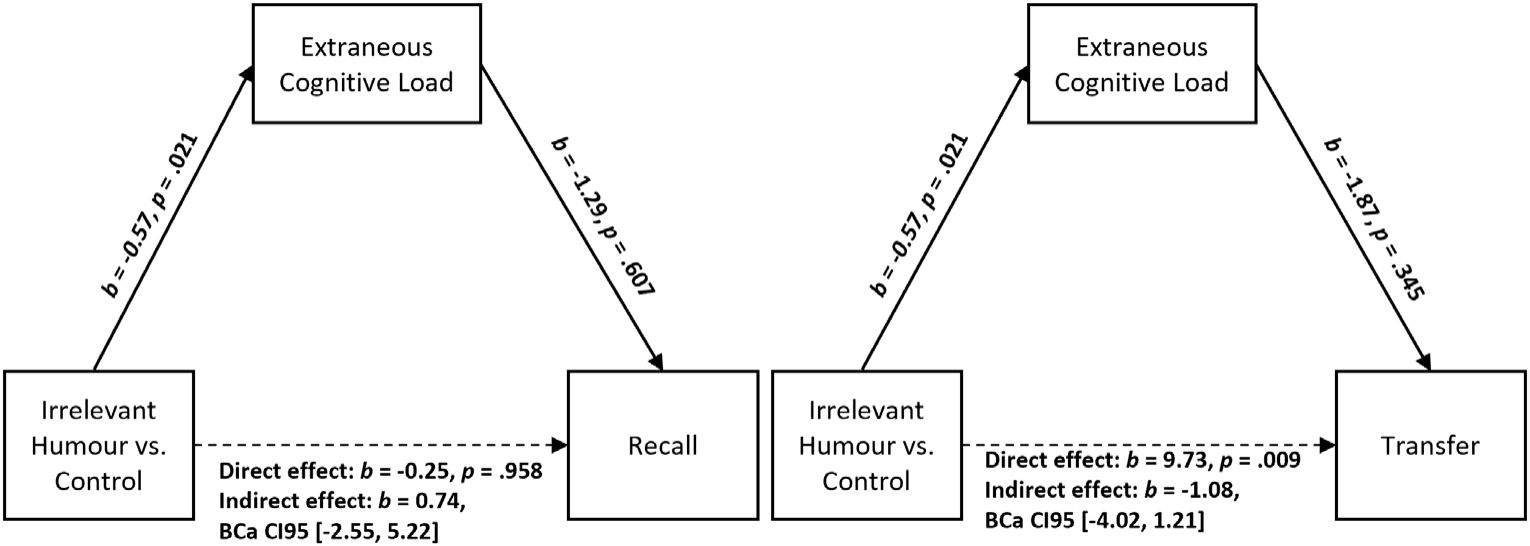
Results of the mediation analysis with condition (control vs. irrelevant humour) as predictor, recall and transfer performance as dependent variable, and extraneous cognitive load as proposed mediator.

A mediation analysis (Hayes, 2022) served to test whether the detrimental effects of irrelevant humour compared to the control group would be mediated by higher extraneous cognitive load ratings (H2.1). Although our analyses revealed significantly higher extraneous cognitive load ratings in the group with irrelevant humour, this effect did not explain the weaker transfer or recall performance in this group, that is, no indirect effect (i.e., no clear evidence for H2.1, see Figure 4).

A second mediation analysis served to check whether participants in the relevant humour group would deliver higher mental effort ratings compared to the control group, thereby increasing learning performance (H2.2). That analysis revealed neither indirect effects nor significant pathways (i.e., no evidence for H2.2).

#### Research question 3: Test of humour processing assumptions

With respect to the affective‐motivational function of our humorous elements (H3), we conducted a MANOVA with planned contrasts testing whether participants in the humorous groups (relevant and irrelevant) showed higher triggered situational interest (feeling towards the material), greater perceived instructor likability and a better perceived learning atmosphere compared to the groups without humorous elements (contrast code, 1, −1, −1, 1; see Table 1 for descriptive values).

**FIGURE 5:**
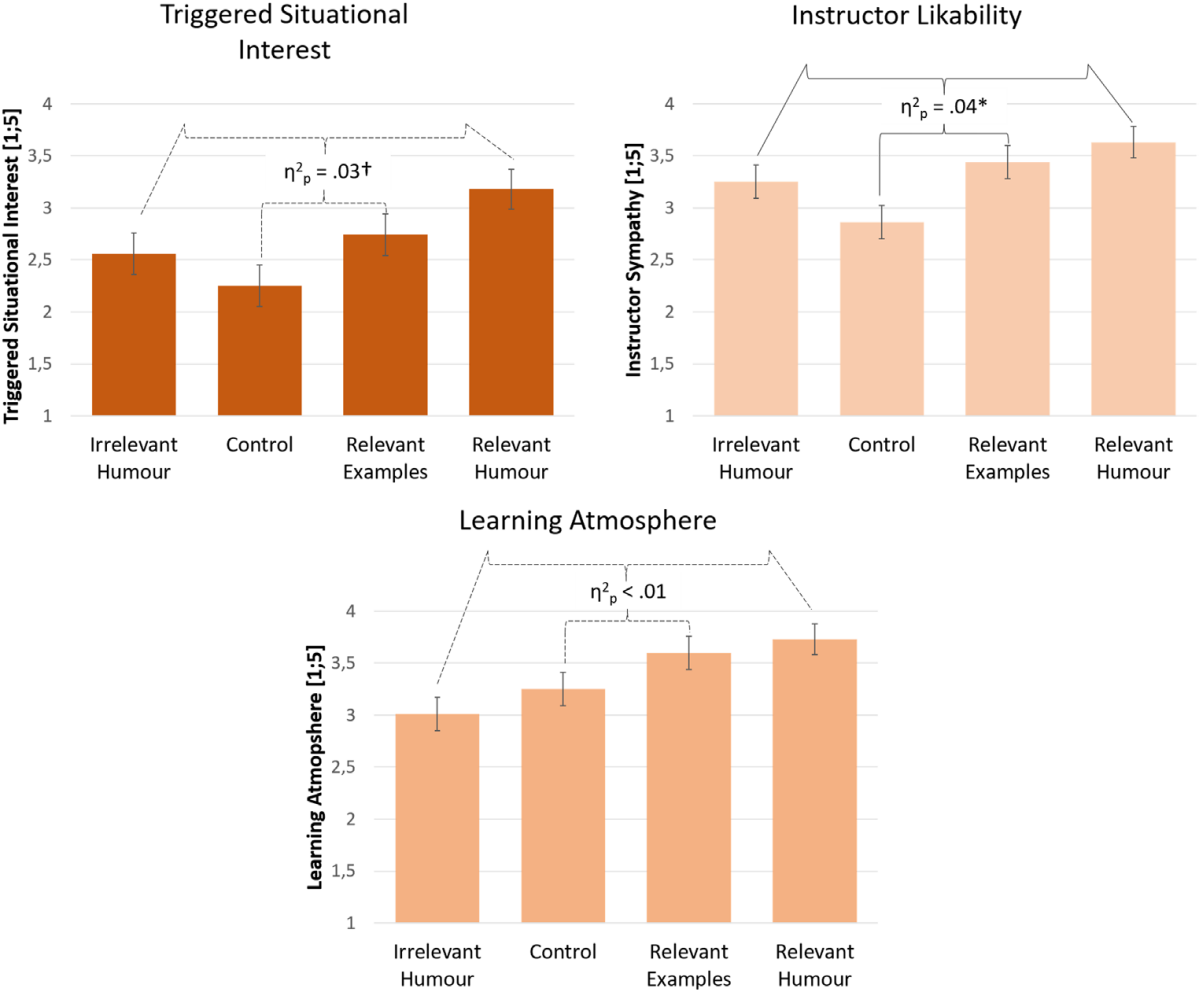
Affective‐motivational variables in the four experimental conditions. Error bars indicate standard errors. ^†^ *p* < .10, *p < .05.

The assumed contrast was significant for participants' perceived instructor likability (*F*(1,111) = 4.61, *p* = .034, ƞ^2^_p_ = .04), but failed slightly to reach statistical significance for triggered situational interest (*F*(1,111) = 3.54, *p* = .063, ƞ^2^_p_ = .03, see Figure 5). Moreover, we failed to observe the assumed pattern of results for learning atmosphere (*F*(1,111) = .12, *p* = .735, ƞ^2^_p_ < .01). In contrast, post hoc analyses revealed significant differences in all three measures between the irrelevant (seductive details) and relevant‐humour group indicating that the affective‐motivational function was stronger in conjunction with relevant humour than with irrelevant (seductive) humour, situational interest: *M*_diff_ = −.62, *p* = .027, 95% CI [−1.17; −.07]; instructor likability: *M*_diff_ = −.38, *p* = .045, 95% CI [−.75; −.01]; learning atmosphere: *M*_diff_ = −.72, *p* = .001, 95% CI [−1.14; −.29]. Hence, our data provided inconsistent evidence with regard to H3.1, H3.2 and H3.3.

Interestingly, the relevant‐examples group rated the instructor's likability as higher than the control group without additional elements (*M*_diff_ = .58, *p* = .003, 95% CI [.20; .96]) and the learning atmosphere as better than in the irrelevant‐humour group (*M*_diff_ = .58, *p* = .009, 95% CI [.15; 1.02]), indicating that the relevant examples, although non‐humorous, might have unintentionally influenced affective‐motivational aspects.

### Discussion of Study 1

#### Research question 1

Overall, our first study indicated that course‐related humour without a cognitive function (i.e., irrelevant humour) leads to worse performance than humour with a cognitive function (i.e., relevant humour) in terms of transfer. Moreover, in line with the *seductive‐humour hypothesis* (H1.1), the study provided evidence that irrelevant humour represents seductive humour, since, compared to no humour, it hampers learning instead of improving it. Hence, irrelevant instructional humour seems to carry the risk of a seductive details effect. We obtained this result only for transfer performance, which (similar to previous seductive details research) indicates that the humour hampered higher cognitive processes specifically.

In contrast to the *effective humour hypothesis* (H1.2), we uncovered no evidence of the benefits of relevant humour compared to no humour. Instead, our results supported the null hypothesis. This finding thus joins the series of inconsistent results from past studies, leaving unanswered the question of when course‐related instructional humour is effective (e.g., Bolkan et al., 2018; Cazes et al., 2024). One possible explanation for the lack of positive effects is that participants—contrary to what was intended—might not have perceived and processed the relevant humorous elements as such (i.e., less perceived relevance; see Eitel et al., 2019). Another possibility is that the motivational demands of our learning unit were too low (e.g., because the learning unit was rather short and well‐structured), so that no additional motivation through humorous elements was necessary. In our second study, we considered those possibilities.

#### Research question 2

In line with our expectations of H2.1, extraneous cognitive load ratings in the group with irrelevant humour were higher than those of the control group. Interestingly, this increase in extraneous cognitive load did not explain the learning decrements, which left the underlying processes indistinct. Moreover, although the relevant humorous elements did improve affective‐motivational instructional aspects, they failed to improve mental effort, thereby contradicting our assumptions of H2.2.

#### Research question 3

We obtained inconsistent evidence regarding H3. Unexpectedly, our data indicated that the affective‐motivational function was stronger in conjunction with relevant humour than with irrelevant (seductive) humour. Moreover, we obtained no clear evidence for an increase in the affective‐motivational dimension for irrelevant (seductive) humour compared to the control group without additional elements. This finding brings up the question of whether irrelevant instructional humour does indeed evoke desired positive effects on affect and motivation. It is therefore difficult to draw clear conclusions about the instructional humour processing theory's affective‐motivational assumptions. In our second study with a different sample and learning material, and an additional measure of perceived relevance, we aimed to get deeper insights concerning this ambiguity.

## STUDY 2

In Study 2, we used the same design as in Study 1, but with the aim of making the potential social–emotional and motivational effects proposed in the instructional humour processing theory more observable. We therefore used a longer learning unit, increasing the required effort (see Endres et al., 2020). Additionally, we conducted the study with a younger sample in a school context, because in previous studies on the seductive details effect, positive affective‐motivational effects were observed specifically in younger students (average age of 13–15 years; e.g., Magner et al., 2014; Lenzner et al., 2013; Wang & Adesope, 2016). It is therefore possible that also the affective‐motivational effects of humour are more pronounced in this age group. One reason for this difference could be lower self‐control abilities in adolescent compared to adult learners, which make them more responsive to gratifying elements in learning materials (see Endres, Bender, et al., 2025; Endres, Vössing, et al., 2025; Luna et al., 2004). Younger learners may even tend to expect entertaining elements in learning materials, as such elements are more common in materials for such age groups (Pozzer & Roth, 2003). As a third measure, we added a talking head to the slide show to create a stronger social presence (Sondermann & Merkt, 2023). Lastly, we also increased the power of our study (see Methods).

As a change to the measures, we added a scale for perceived relevance of the additional jokes/examples to test whether they were indeed perceived as intended. To that, we added a measure of existing interest as a control variable. Due to classroom time constraints and the lack of appropriate measures, we were unable to assess spatial abilities in this study, but instead relied on students' latest geography grade as an indicator of cognitive prerequisites (we also conducted robustness checks without the covariates for both studies).

### Participants, design and material

Due to the inconsistent effects in study 1, we decided to increase the power in this study to 90%. The respective a‐priori power‐analysis revealed a required sample size of 139 participants to detect a statistically significant linear contrast in a contrast analysis (directed hypothesis, α‐level *p* = .10, Cohen's *f* = .25, power 90%). Again, we considered potential drop‐outs and therefore collected data from 158 students in the 6th and 7th grade at a German high school. We excluded two participants from our analyses due to technical issues. Furthermore, in line with our study requirements, two participants were excluded because their German language skills were not at the required level, and another three participants were excluded because they said they had dyslexia, which could have influenced their answers on the self‐rating and post‐test items. Our final sample numbered 151 participants (*M*_age_ = 12.21, SD = .76).

**FIGURE 6:**
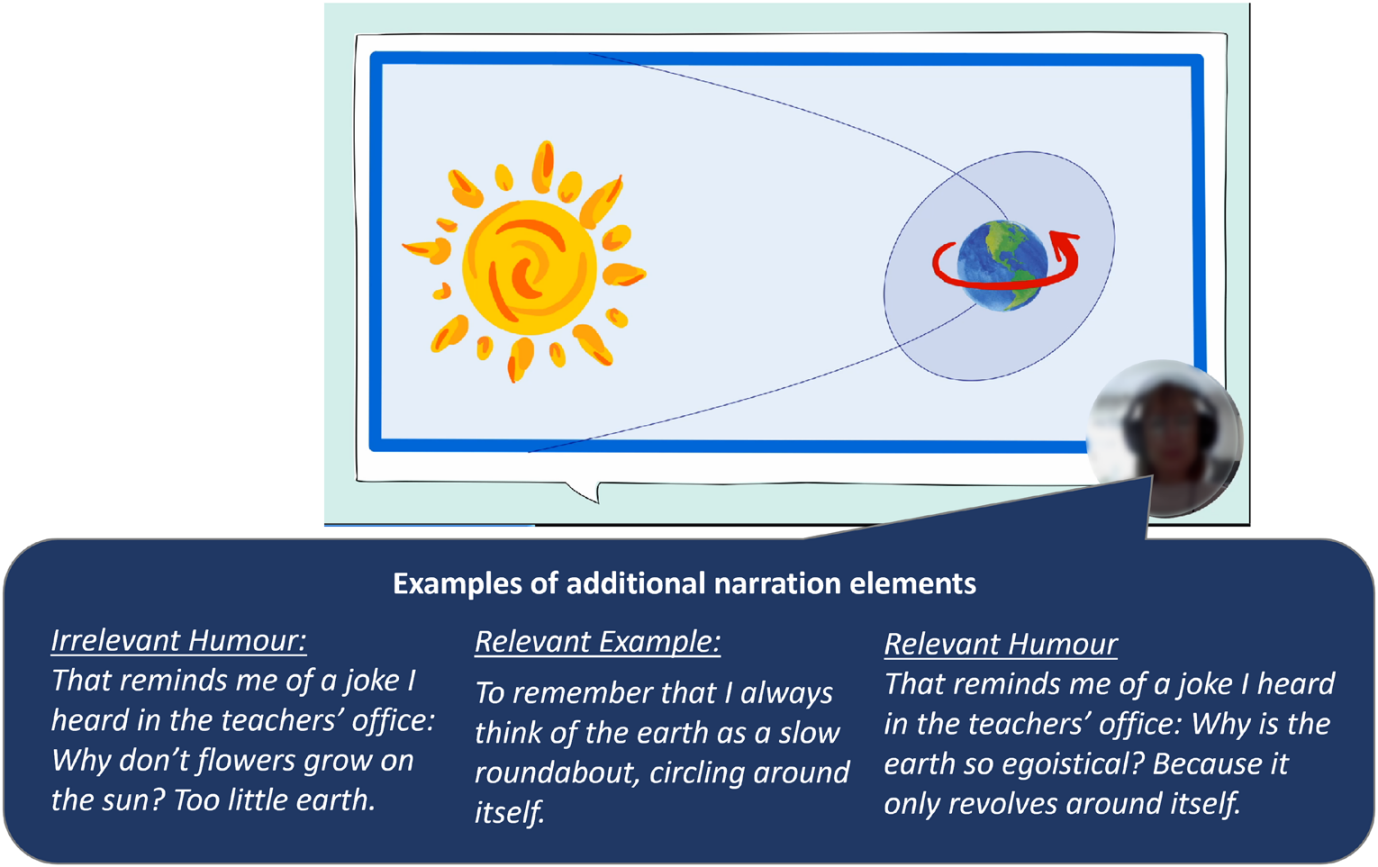
Screenshot of the learning material (narrated slides) and the additional narration elements in the four experimental groups of study 2.

This study followed the same design as in study 1 (see Figures 1 and 6). The learning material for this study comprised a newly developed narrated slide show about solar and lunar eclipses. The narrated text was based on texts from children's encyclopaedias, websites and journals and was of appropriate complexity for our age group (German version of Flesch–Kincaid score = 37.04; Lenhard & Lenhard, 2014). The slides consisted of 16 partially animated schematic illustrations in which we highlighted the important components with circles and arrows. The slideshow also included the talking head of a middle‐aged female narrator on the bottom right whose facial expressions were mild (e.g., slight smiles), but there were no gestures. The talking head was visible in the lower right corner. The base video lasted 11 min 43 s and could not be paused by participants.

In three of our four experimental groups, the oral narration contained eight additional narrated elements according to the experimental manipulation (see Figure 6 and the study 1 in the Methods section). The additional elements represented (only) 17% of the total narration to keep the learning unit from appearing ridiculous or absurd. The experimental videos were between 30 s and 2 min longer than the base video.

We tested the jokes and examples regarding their funniness and cognitive function in a sample of 19 participants. A repeated‐measures contrast analysis revealed that participants rated the relevant (*M* = 4.04, SD = .45) and irrelevant jokes (*M* = 3.99, SD = .60) as funnier than the relevant examples (*M* = 2.42, SD = .92), *F*(1,18) = 31.62, *p* < .001, ƞ^2^_p_ = 64. Moreover, they found that the relevant jokes (*M* = 3.66, SD = .74) and relevant examples (*M* = 3.70, SD = .76) promoted understanding more than the irrelevant jokes (*M* = 1.66, SD = .73), *F*(1,18) = 108.71, *p* < .001, ƞ^2^_p_ = 86.

### Measures

Unless indicated otherwise, all Likert self‐rating scales ranged from 1 ‘strongly disagree’ to 5 ‘strongly agree’.

#### Prior knowledge

To control for cognitive prerequisites, we assessed participants' last grades in geography/earth science and physics/natural sciences. Moreover, five self‐rating items (z‐standardized; Cronbach's α = .53) and four knowledge items (Cronbach's α = .09) served to check subject‐specific prior knowledge. Due to the low internal consistency of the knowledge items, we refrained from conducting any further analysis with this scale.

#### Existing interest

As a further control variable, we assessed participants' existing interest in the topic of solar and lunar eclipses prior to learning (Hidi & Renninger, 2006). Participants rated nine items (feeling, value and reengagement) on a 5‐point Likert scale (e.g., ‘I think solar and lunar eclipses are very interesting.’, Cronbach's α = .87; Endres, Vössing, et al., 2025).

#### Cognitive load and mental effort

We assessed cognitive load with the same scale as in study 1 (Klepsch et al., 2017; α_intrinsic_ = .54, α_extraneous_ = .73, α_germane_ = .58). To assess mental effort, we used three items of the scale by Endres, Bender, et al. (2025); e.g., ‘I worked hard on this activity.’, 5‐point Likert scale; (Cronbach's α = .83). Due to a theoretical and empirical overlap between germane cognitive load and mental effort (*r* = .65, *p* < .001; Endres, Bender, et al., 2025; Klepsch & Seufert, 2021), we again computed a composite score, henceforth called mental effort (Cronbach's α = .83).

#### Situational interest, likability and atmosphere

The same scales as in study 1 served to assess situational interest (α_triggered_ = .86; α_maintained_ = .88), instructor likability (α = .70) and atmosphere (α = .79) retrospectively after the learning unit.

#### Perceived relevance

After the learning unit, participants also answered three items assessing their perceived relevance of the additional relevant and irrelevant elements in the three experimental conditions on a scale from 0% to 100% with five gradations (e.g., ‘How relevant to the learning content did you find the jokes/examples?’, Cronbach's α = .73; adapted from Bender et al., 2021a).

#### Learning performance

To keep our young participants from becoming demotivated, we ran a mixed‐format test instead of a free recall task to measure recall of the learning content (4 open‐ended and 6 closed‐format questions). Correct retention and basic comprehension of the main aspects were required for high scores on this test (e.g.: ‘Please explain why the sun and moon appear to be equally large in the sky.’). An additional six questions (1 open‐ended question and 5 multiple‐choice questions) served to assess transfer performance (e.g.: ‘What would people see if they were standing on the moon and looking towards the earth during a lunar eclipse?’).

We applied the same scoring procedure as in study 1 (21% of answers double‐coded), resulting in good interrater‐reliability ICC_2,1_ = .98 (two‐way random effects, absolute; Shrout & Fleiss, 1979). For the multiple‐choice questions, we allocated one point for each correctly chosen answer option. With Cronbach's α = .59 for the recall scale and Cronbach's α = .62 for the transfer scale, we obtained acceptable internal consistency for heterogeneous constructs (Schmitt, 1996).

### Procedure

This experiment was conducted in class sessions in the computer room at a German high school (one class per session, 22 to 27 students). Participants completed the study individually on the computer. Before initiating the study, we obtained consent and approval from the parents and the government. We again asked for participants' consent at the beginning of the study. Apart from that, the procedure and instructions were similar to study 1. The sessions lasted about 35 min.

### Results

#### Research question 1: Learning outcomes

**FIGURE 7:**
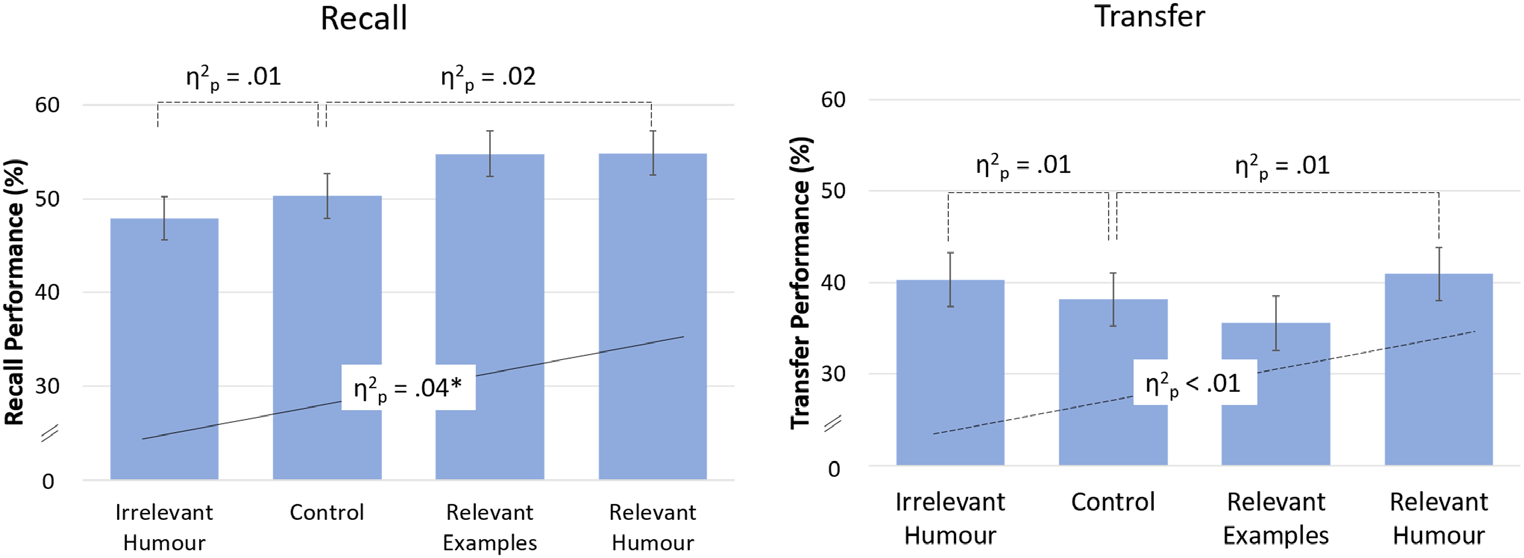
Recall and transfer performance in the four experimental conditions as a function of the latest geography grade. Error bars indicate standard errors. *p < .05.

See Table 2 for descriptive values. To test our hypothesized pattern of results, we conducted a MANCOVA with a planned linear contrast. Recall and transfer performance served as dependent variables. The latest geography grade was used as control variable due to moderate correlations with recall (*r* = −.28) and transfer (*r* = −.25).^3^ The linear contrast test was not significant for transfer, *F*(1,146) = .07, *p* = .789 and ƞ^2^_p_ < .01, but revealed a significant linear trend for recall performance, *F*(1,146) = 5.93, *p* = .016 and ƞ^2^_p_ = .04 (see Figure 7).

**TABLE 2:** Means (and standard deviations) of the main variables in the four experimental groups and condensed over all groups of study 2.

	Irrelevant humour	Control	Relevant examples	Relevant humour	Overall
Recall %	47.60 (17.86)	51.39 (13.89)	54.42 (14.57)	54.45 (13.29)	51.95 (15.12)
Transfer %	39.95 (17.78)	39.47 (15.39)	35.10 (19.64)	40.44 (21.14)	38.80 (18.55)
ECL [1; 5]	2.50 (.84)	2.32 (.76)	2.19 (.71)	2.36 (.73)	2.34 (.76)
ICL [1; 5]	3.01 (.71)	2.93 (.87)	2.79 (.75)	2.94 (.79)	2.92 (.78)
Mental effort [1; 5]	3.56 (.61)	3.40 (.82)	3.24 (.94)	3.64 (.69)	3.46 (.78)
Triggered situational interest [1; 5]	3.14 (.82)	3.15 (.92)	2.95 (1.04)	3.19 (1.05)	3.11 (.96)
Maintained situational interest [1; 5]	3.63 (.70)	3.57 (.82)	3.42 (.88)	3.70 (.75)	3.58 (.78)
Instructor likability [1; 5]	3.69 (.75)	3.37 (.68)	3.28 (.68)	3.60 (.66)	3.49 (.70)
Learning atmosphere [1; 5]	3.53 (.62)	3.30 (.70)	3.22 (.83)	3.49 (.76)	3.39 (.73)
Perceived relevance of additional elements [1; 5]	2.12 (.77)	–	3.57 (.75)	2.30 (.91)	–
Existing interest [1; 5]	2.96 (.68)	2.84 (.68)	2.89 (.74)	3.20 (.79)	2.97 (.73)
Prior knowledge (z‐standardized)	−.06 (.58)	−.02 (.49)	.02 (.65)	.05 (.64)	.00 (.59)

Abbreviations: ECL, extraneous cognitive load; ICL, intrinsic cognitive load.

With regard to the *seductive‐humour hypothesis* (H1.1), post‐hoc comparisons showed that the irrelevant‐humour group performed significantly worse than the relevant humour (*M*_diff_ = −6.96, *p* = .036, 95% CI [−13.46; −.47]) and the relevant‐examples group (*M*_diff_ = −6.89, *p* = .042, 95% CI [−13.52; −.36]) in the recall test. However, there was no significant difference to the control group for recall, *M*_diff_ = −2.40, *p* = .473, 95% CI [−8.98; 4.18], or transfer performance, *M*_diff_ = 19.54, *p* = .622, 95% CI [−58.67; 97.75] (i.e., no clear evidence for H1.1). Following up on this lack of a significant difference to the control group, we additionally conducted Bayesian ANCOVAs (irrelevant humour vs. control) to test the null hypothesis. The Bayes analysis revealed moderate evidence favouring the null hypothesis for both recall (BF_01_ = 3.45) and transfer (BF_01_ = 3.59) performance.

In contrast to the *effective humour hypothesis* (H1.2), participants in the relevant‐humour group did not outperform the control group in the recall, *M*_diff_ = 4.57, *p* = .170, 95% CI [−1.98; 11.11] or transfer test, *M*_diff_ = 14.28, *p* = .717, 95% CI [−63.53; 92.08]. Again, we additionally conducted a Bayesian ANCOVA (relevant humour vs. control). The Bayes analysis revealed anecdotical evidence for recall (BF_01_ = 2.29) and moderate evidence for transfer (BF_01_ = 3.70) performance favouring the null hypothesis.

#### Research question 2: Cognitive load and mental effort

Four mediation analyses testing our hypotheses regarding extraneous load and mental effort did not reveal any indirect effect (see data set on OSF for detailed results). Hence, we obtained no evidence for H2.1 and H2.2.

#### Research question 3: Test of humour processing assumptions

**FIGURE 8:**
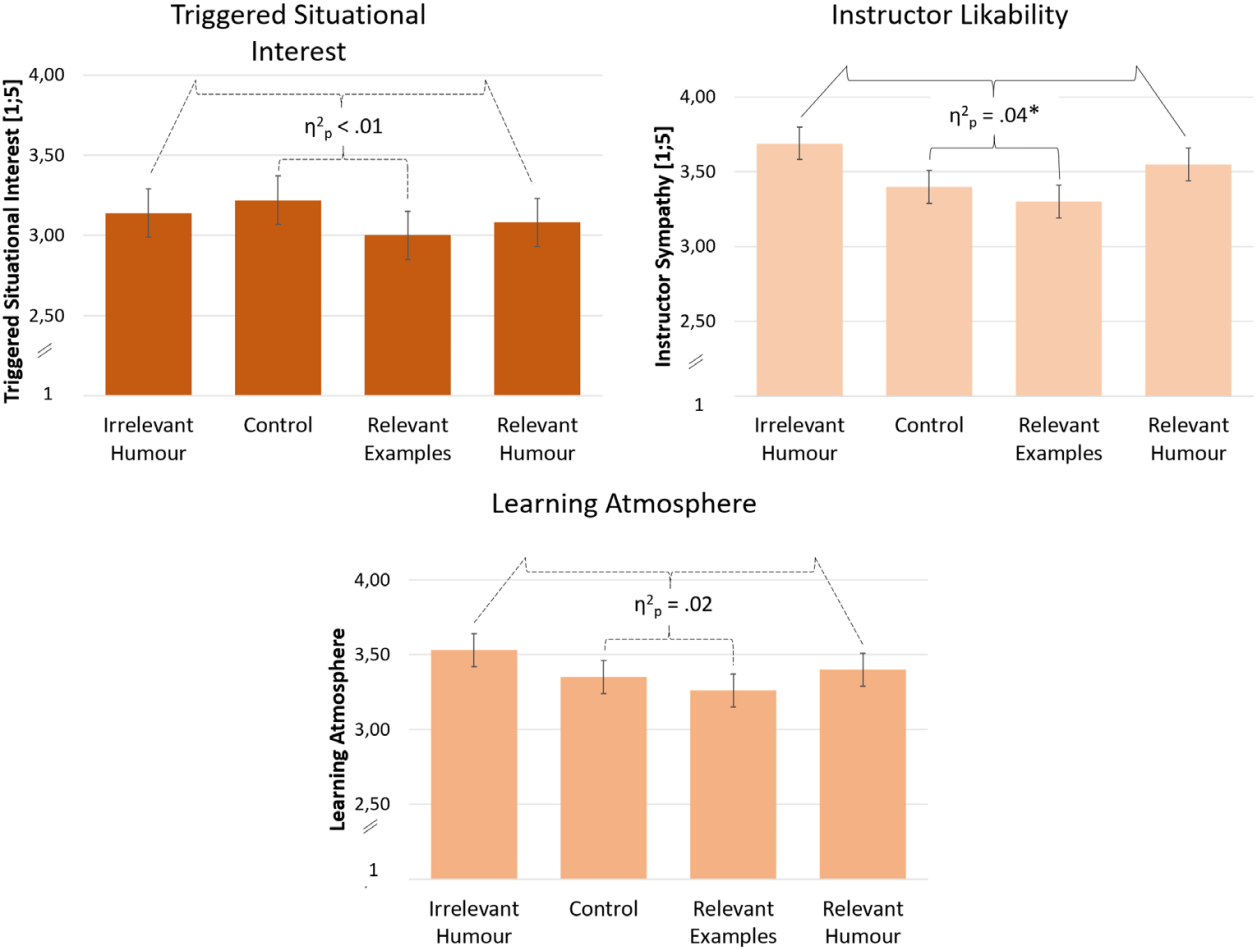
Affective‐motivational variables in the four experimental conditions as a function of existing interest. Error bars indicate standard errors. *p < .05.

Again, we checked the affective‐motivational function of our humorous elements via a MANCOVA with planned contrasts controlling for existing interest. In line with H3.2, the contrast analysis revealed that participants in the two humorous groups perceived instructor likability (*F*(1,146) = 5.65, *p* = .019, ƞ^2^_p_ = .04) as higher than the groups without humorous elements (contrast code, 1, −1, −1, 1; see Figure 8). The contrast was, however, not significant for learning atmosphere (*F*(1,146) = 1.92, *p* = .168, ƞ^2^_p_ = .01) and triggered situational interest (*F*(1,146) = .00, *p* = .994, ƞ^2^_p_ < .01; i.e., no evidence for H3.1 and H3.3). There were no further significant differences in either of the dependent variables.

#### Perceived relevance

In addition, we checked whether participants in the relevant humour and examples groups perceived the additional elements indeed as more relevant than participants in the irrelevant humour (seductive details) group. A contrast analysis revealed a significant main contrast comparing the two relevant groups with the irrelevant group (*F*(1,110) = 25.21, *p* < .001, ƞ^2^_p_ = .19), that is, participants in relevant groups actually perceived higher relevance. However, post‐hoc comparisons revealed a significant difference between the two relevant groups with each other (*M*_diff_ = 1.28, *p* < .001, 95% CI [.90; 1.65]), with higher perceived relevance in the relevant examples than in the relevant‐humour group. In contrast, the post‐hoc comparison between the irrelevant and relevant‐humour group showed no significant difference (*M*_diff_ = −.18, *p* = .344, 95% CI [−.54; .19]). Hence, our results indicate that only relevant examples (but not relevant jokes) were clearly perceived as more relevant than irrelevant jokes.

### Discussion of Study 2

#### Research question 1

In study 2, we made some changes to the sample and material to raise the probability of detecting relevant humour's potentially positive effects. As in Study 1, we observed that the group exposed to irrelevant humour performed worse than the group exposed to relevant humour, this time in their recall performance. This difference was accompanied by the assumed significant linear trend. Hence, again, we have gathered evidence of the superiority of relevant humour over irrelevant humour. However, our comparison to the control group of these two kinds of humour was less conclusive: We obtained neither evidence for the seductiveness of irrelevant humour (i.e., no significant detrimental effects; H1.1), nor evidence for the effectiveness of relevant humour (i.e., no significant positive effects; H1.2) compared to no additional elements. Rather our data tentatively supported the null hypotheses.

#### Research questions 2 and 3

We obtained no evidence for H2.1 and H2.2, that is, no effects on cognitive load and mental effort. Moreover, the results regarding affective‐motivational aspects were again inconclusive. While we found the assumed higher instructor likeability ratings in the two humorous groups (H3.2), no such effects were present for situational interest (H3.1) and learning atmosphere (H3.3), leaving the actual effects on a processing level somewhat unclear. Interestingly, this study provided tentative evidence that perceived relevance might be influenced by the humour aspect, because we identified less perceived relevance of relevant humour compared to relevant examples. This finding may constitute—at least in part—one explanation for the inconsistent effectiveness of relevant humour, which we discuss below.

## GENERAL DISCUSSION

We all experience humour many times a day in various forms and contexts. Our research aimed to advance the question of when humour in instructional contexts is effective for learning and when it is ineffective instead. For this purpose, we considered research on instructional humour as well as research from the related field of seductive details. In the following, we discuss potential negative (*seductive humour hypothesis*) and positive (*effective humour hypothesis*) effects of instructional humour, thereby integrating the answers to our research questions 1–3 for each direction of effects.

### Seductive‐humour hypothesis

With respect to our first research question, both of our studies indicated that instructional humour is ineffective when it serves no cognitive function (i.e., does not facilitate the processing of relevant content). More specifically, in study 1's adult sample, irrelevant humour resulted in a worse transfer performance compared to relevant humour and no humour (i.e., evidence for the *seductive‐humour hypothesis*, H1.1). Moreover, partly in line with H2.1, we observed an increased extraneous cognitive load in this group compared to the control group. Those findings concur with seductive details research (e.g., Eitel et al., 2019; Schneider et al., 2019) and indicate that irrelevant instructional humour could indeed cause similarly detrimental cognitive processes as do ‘classical’ seductive details (i.e., interesting but irrelevant facts and/or pictures).

Interestingly, in study 2's school sample, irrelevant humour was also detrimental compared to relevant humour, but we observed no significant difference from the control group without humour (i.e., no consistent evidence for the *seductive‐humour hypothesis*, H1.1). One possible reason for this finding is that the learning material in study 2 contained fewer jokes (30% in study 1 vs. 17% in study 2). Since the seductive details effect in past studies was usually apparent when around 30% of the learning material contained seductive details, this finding may be an interesting avenue to pursue in future research (Harp & Mayer, 1998; Wang et al., 2021). An alternative explanation for the inconsistency of the negative effect could lie in the sample's age. Previous studies investigating seductive details with younger samples yielded fairly inconsistent results (Lenzner et al., 2013; Magner et al., 2014; Wang & Adesope, 2016), and we could therefore assume that irrelevant humorous or interesting elements in a lesson may exert different effects on adults than on children or adolescents. For example, it is possible that younger learners are more used to entertaining elements in their learning materials than are adult learners and therefore enjoy them less when having to learn without such elements (e.g., Pozzer & Roth, 2003; Roth et al., 1999). Consequently, irrelevant humour might satisfy the younger students' needs by counteracting potentially detrimental cognitive effects. Since such differences would provide important practical implications, further studies should aim to test systematically these effects in different age groups.

Regarding irrelevant humour's affective‐motivational effects (i.e., H3.1 to H3.3), we observed inconclusive evidence. In both studies, irrelevant humorous elements increased perceived instructor likability, but had no clear effect on situational interest or the learning atmosphere compared to the conditions without humour. This finding is also in line with inconsistent evidence on the affective‐motivational effects of seductive details (Bender et al., 2021a). Overall, it may simply be very difficult to evoke the desired (measurable) effects on students' affect and motivation. We consider this finding to be particularly important for real‐life educational contexts, as it challenges the widespread intuitive assumption that jokes, anecdotes or fun facts are a generally suitable way of motivating students and improving the learning atmosphere. Since we did find effects on instructor likability in both studies, it is possible that such irrelevant jokes may serve primarily as a social lubricant (e.g., reducing tension, strengthening the relationship between teachers and students). Especially over longer time periods, such effects might eventually also increase students' motivation and effort.

### Effective humour hypothesis

Our studies revealed rather inconsistent evidence with respect to the *effective humour hypothesis* (H1.2). Both our studies yielded evidence that humour might be more effective when it is relevant rather than irrelevant. However, we observed no significant differences from the condition without additional elements (control). Moreover, despite the higher ratings of situational interest (H3.1) and learning atmosphere (H3.3) in study 1 and higher instructor likability in both studies (H3.2), relevant humour did not lead to increased mental effort ratings (H2.2). Interestingly, descriptive values and a significant linear contrast in study 2's recall measure still provided tentative evidence supporting our expectations of H1.2, indicating that positive effects might be more likely observed in a younger sample and/or longer learning session (Endres et al., 2024). This finding provides avenues for future research aiming to investigate the positive effects of course‐related humour.

Nevertheless, considering our results and previous inconsistent findings (e.g., Bolkan et al., 2018; Cazes et al., 2024), we can come to no clear conclusion with respect to the effectiveness of using relevant humour in either age group. Our results, therefore, fail to clearly support the assumptions of the instructional humour processing theory (Wanzer et al., 2010). We therefore encourage more investigations applying similar designs and/or considering different mediators and moderators.

One interesting avenue could lie in the perceived relevance of the additional elements measured in study 2. Those analyses revealed that participants in the relevant humour condition rated the additional elements as less relevant than those in the relevant examples condition (i.e., relevant jokes were perceived as less relevant than relevant examples). This finding may indicate that—although the jokes and examples were equally relevant—students in the relevant jokes condition processed the jokes less thoroughly and effortfully than students in the relevant examples condition. This difference, consequently, would mean that the relevant jokes diminished potentially positive effects. It would therefore be important to determine whether presenting relevant content humorously tends to carry the risk of reducing the perceived importance or relevance of this content (e.g., due to processing heuristics or overconfidence; see e.g., Senko et al., 2021; Um et al., 2012). Among others, such an idea could be tested by fully crossing the two factors humour (non‐humour vs. humour) and relevance (irrelevant vs. relevant), thereby allowing for a test of the main effect of humour on perceived relevance. This design would also be interesting in terms of main and interaction effects on learning performance.

### Limitations

The first important limitation concerns our experimental scope. As the effects of instructional humour are complex, manifold and appear on different levels, our studies can only reflect a part of the picture. Future studies should examine the long‐term effects, the effects of different humour types and placements, and the social effects in more detail (see, e.g., survey studies by Banas et al., 2011; Bieg et al., 2019). From our results, we consider especially the (long‐term) social effects to be a promising path to follow because even in our short instructional videos without a physically present instructor, we observed an effect on ratings of instructor likability.

The second limitation refers to the (partial) lack of effects on affective‐motivational outcomes. Although we tested our materials for their entertainment value, the jokes did not prove to robustly improve affective‐motivational outcomes. Moreover, in study 1, we even observed an unintended increase in those measures for relevant examples compared to the control group. Both these factors may indicate that our manipulation did not work as intended. While we still consider this an important finding with regard to real‐life contexts where such inconsistent effects can also be observed, we still encourage replication studies. Important in research and practice is the fact that a difficulty also arises from humour's relative subjectivity, which could make the same joke funny to some students but irritating to others (see Martin et al., 2003). The perception of humour might be very specific to factors such as gender, age, or cultural background. It is unclear how well our pilot samples matched the samples of the main study with regard to those characteristics since we did not collect any personal data in the pilot tests. In study 2, in particular, the pilot sample may differ from the main study sample in terms of age. In future studies, it would be sensible to better match the pilot and the study samples.

A third limitation is that in study 1, we only detected effects on transfer performance, whereas in study 2, the effects occurred only in recall performance. This result pattern may be attributable to differences in the learning materials' complexity (e.g., because study 2's learning material was longer). Importantly, our recall measure also comprised a basic understanding of central concepts, which is why we tend to assume that the humorous elements influenced the comprehension performance as well (not solely retention).

Lastly, in both studies, we assessed self‐rated (subjective) prior knowledge. We had no (reliable) objective measure of prior knowledge. The interpretability of such subjective measures is limited, as (especially younger) students often struggle to accurately self‐assess their knowledge (Finn & Metcalfe, 2014). Future studies should employ objective prior knowledge tests (e.g., via free production tasks). Such a test would allow us to examine the potentially moderating effects of prior knowledge (Fries et al., 2019).

### Implications

Our studies indicate that instructional humour carries the risk of a seductive details effect. Irrelevant humour (even when related to the content) could impede the learning process. Note, however, that it would be presumptuous to assume that all irrelevant humour must be avoided in an educational context. Our research especially refers to occasions where instructors purposefully tell jokes or funny anecdotes during direct instruction (e.g., a lecture or expository video). In such situations, we recommend the cautious and deliberate use of humour. Not only might irrelevant humour during direct instruction impede cognitive processing, but it may also fail to have the intended positive affective‐motivational effects. Replication studies are, however, needed in order to check whether our results are robust for other materials and manipulations. The findings of our study are also important with regard to the investigation by Abbott and Jones (2003). Their survey revealed that—although according to student and teacher reports, *course‐related* humour is used most frequently (Bieg et al., 2019)—73% of observed teacher humour was indeed *unrelated* to the course content (see Robinson et al., 2024 for an overview). It therefore seems overall desirable for instructors to be more aware of the humour they use and its effects.

We can come to no clear conclusions regarding relevant humour. If instructors want to use humour, our results indicate that it is better when such humour serves a cognitive function, but we cannot claim that relevant humour per se does indeed help learning (compared to no humour). As stated above, our studies only cover certain aspects of humour's manifold effects and facets in educational settings and it is unclear whether our manipulations had the intended effects, but given the overall inconclusive evidence, it might be safer to use humour as a social lubricant during pauses or social activities instead of incorporating it specifically in the instruction.

Lastly, the absence of a negative effect of irrelevant humour in our younger sample may contribute to developing the theory on the seductive details effect, as it underlies previously identified tendencies in result patterns. As mentioned above, we consider further investigation of this tentative finding to be a worthwhile approach.

## CONCLUSION

Our studies provided tentative evidence on when humour in instructional settings might be effective or ineffective. Overall, we conclude that irrelevant humour—even when related to the learning content—carries the risk of impeding learning. More research is needed to come to clear conclusions about relevant humour.

## AUTHOR CONTRIBUTIONS

**Lisa Bender:** Conceptualization; investigation; writing – original draft; data curation; visualization; formal analysis; project administration; software; methodology. **Alexander Renkl:** Conceptualization; writing – review and editing; supervision; resources; validation. **Tino Endres:** Conceptualization; writing – review and editing; supervision; investigation; methodology; validation.

## CONFLICT OF INTEREST STATEMENT

The authors declare no conflict of interests.

## Data Availability

The data that support the findings of this study are openly available in Open Science Framework at https://osf.io/gnkf9.

## Footnotes

^1^
As intrinsic cognitive load and maintained situational interest were not directly related to our hypotheses, we decided to refrain from reporting any respective analyses in the present article. Nevertheless, the data are available on OSF under the following link: https://osf.io/gnkf9.

^2^
We obtained a similar result pattern when conducting the analyses without the covariate (see OSF).

^3^
We received a similar pattern of results when conducting the analyses without the covariate (see OSF).
